# Determination of Inorganic Ions in Parenteral Nutrition Solutions by Ion Chromatography

**DOI:** 10.3390/molecules27165266

**Published:** 2022-08-18

**Authors:** Zhiqi Wen, Kris Wolfs, Ann Van Schepdael, Erwin Adams

**Affiliations:** Pharmaceutical Analysis, Department of Pharmaceutical and Pharmacological Sciences, KU Leuven, 3000 Leuven, Belgium

**Keywords:** parenteral nutrition, sample pretreatment, inorganic ions, ion chromatography

## Abstract

A new, simple and sensitive ion chromatography (IC) method for the determination of sodium, potassium, magnesium, calcium and chloride in a parenteral nutrition (PN) solution was developed and validated. Before sample analysis, a sample pretreatment by calcination was applied which could totally remove interference from other constituents of the PN solution. Methanesulfonic acid (MSA) and sodium hydroxide were used as the mobile phase for the determination of cations and anions, respectively. The calibration curves showed good correlation between analyte peak area and concentration (r^2^ > 0.999). Detection limits ranged from 0.0001 to 0.02 mg/L and quantification limits from 0.0002 to 0.06 mg/L. Relative standard deviation (RSD) values for repeatability and inter-day precision did not exceed 1.0% and the recoveries for all analytes were between 99.1–101.1%. The robustness was verified by using an experimental design.

## 1. Introduction

Parenteral nutrition (PN) is administered intravenously and has been applied in clinical practice for over 50 years [1]. Currently, PN is used in several therapies of patients with short bowel syndrome, gastrointestinal bleeding, bowel obstruction and absorption disorders [2]. It also provides nutrition support for cancer patients, geriatric populations and preterm infants who have poor nutrition intake [3,4,5]. As PN is mainly used for feeble patients, the compounding of PN solutions in hospital pharmacies needs a strict control for quality assurance [6]. PN solutions usually contain over 20 ingredients, including electrolytes, glucose, amino acids and trace elements, and these nutritional solutions are typically prepared in hospital pharmacies. To avoid the risk for patients caused by errors in the electrolyte concentrations, quality control should be performed but is often limited [6]. Currently, sodium, potassium, magnesium and calcium are usually analyzed by potentiometric or photometric/spectrometric methods [7]. However, reproducibility of these methods is rather poor, they can be labor intensive, and the large amount of amino acids in PN solutions may interfere with the determination of the ions [7,8]. Therefore, other analytical techniques are required.

There are some widely used techniques for analyzing inorganic ions, such as atomic absorption spectroscopy (AAS), capillary electrophoresis with capacitively coupled contactless conductivity detection (CE-C^4^D), inductively coupled plasma quadrupole mass spectrometry (ICP-MS) and ion chromatography (IC) [7,9,10,11,12,13,14]. Some of them, such as CE-C^4^D and ICP-MS have been developed to analyze inorganic ions in PN solutions [7,14]. The drawbacks of CE are poor reproducibility of migration times and peak areas, which restrict its use for routine analysis [15]. The widespread use of ICP-MS is limited due to its high cost.

IC is a variant of high-performance liquid chromatography. As a highly sensitive and selective technique for the determination of inorganic ions, it has been used for routine determination of cations and anions in a wide variety of samples in many industries, including environment, biotechnology, agriculture, food and pharmaceutical industries [16,17]. Numerous methods were developed to determine sodium, potassium, magnesium, calcium and chloride utilizing IC with as main benefits compared to other analytical techniques: short analysis time, excellent sensitivity, high selectivity, small sample volume and cost-effectiveness [8,15]. Cation-exchange columns and anion-exchange columns have been applied for the determination of the respective ions due to their affinity to the specific ion exchanger. Conductivity detection is a universal detection technique in IC, including the suppressed and non-suppressed mode. For the suppressed mode, a suppressor is installed after the analytical column to lower the background signal and increase the sensitivity. For the non-suppressed mode, there is only a conductivity detector after the column making the system simpler and cheaper. Even though IC is commonly used in inorganic ions analysis, no study has been found in the literature describing the determination of sodium, potassium, magnesium, calcium and chloride in PN solutions.

In this study, IC methods for the determination of sodium, potassium, magnesium, calcium and chloride in PN solutions were developed using a one-factor-at-a-time approach. This was preferred above a design of experiments because influencing factors (including non-continuous ones) were difficult to predict in advance. Mainly, issues related to the sample pretreatment were encountered and solved step by step. After optimization, the methods were validated.

## 2. Results and Discussion

### 2.1. Method Development and Optimization for Cation Analysis

#### 2.1.1. IC Separation Issues

**Figure 1:**
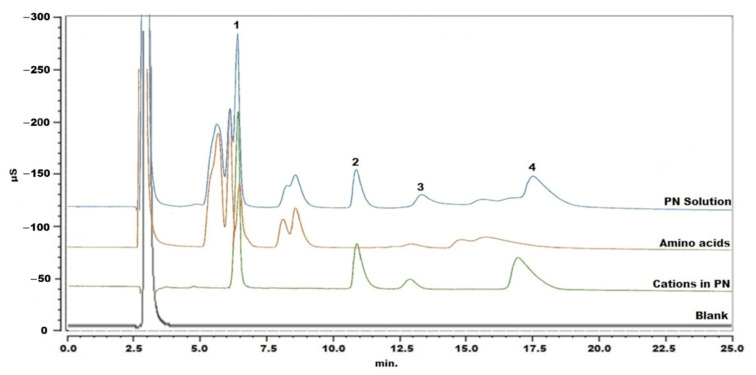
Chromatogram of blank, standard solution of cations in the PN solution, mixture of amino acids and PN solution without pretreatment. Peak 1: sodium, peak 2: potassium, peak 3: magnesium and peak 4: calcium.

As starting conditions, a cation analysis method for column testing was applied: cations were separated with an IonPac CS16 column kept at 40 °C and the mobile phase was 30 mM MSA with a flow rate of 0.36 mL/min [18]. The PN sample was injected following simple dilution with Milli-Q water. The chromatogram showed that the amino acids interfered with the determination of sodium and calcium (Figure 1). Therefore, adjustment of the chromatographic parameters was explored, in an attempt to separate peaks of analytes from interfering components. Different mobile phases were used, including 20 mM MSA, 30 mM MSA, 40 mM MSA, 30 mM MSA with 5% acetonitrile (ACN), 30 mM MSA with 10% ACN, 2 mM MSA with 10% ACN and 30 mM MSA with 5% tetrahydrofuran. A lower concentration of MSA or organic modifier could obviously delay the retention times, but this had limited improvement on the separation of sodium and the interfering substances. In this way, 30 mM MSA was maintained for further experiments. When changing the temperature in steps of 2 °C from 40 °C to 34 °C, peaks shifted only slightly. The best selectivity was obtained at 36 °C, but sodium and calcium ions could not yet be properly determined. In a next step, cleanup of the sample was explored.

#### 2.1.2. Cleanup Procedure

**Figure 2:**
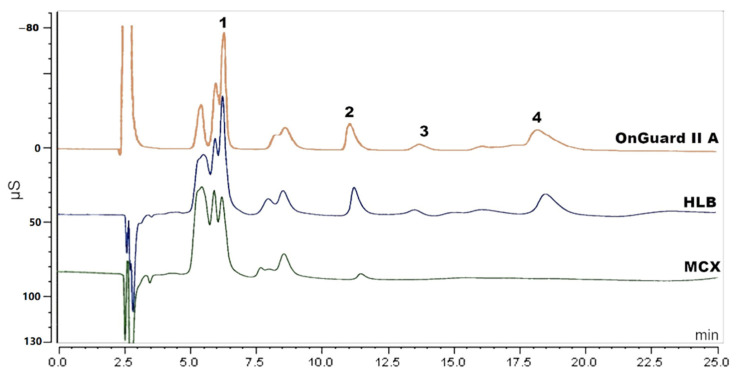
Chromatogram of sample solution pretreated by MCX, HLB and OnGuard II A cartridge. Peak 1: sodium, peak 2: potassium, peak 3: magnesium and peak 4: calcium.

Solid-phase extraction (SPE) has been reported to extract amino acids [19,20]. Three different SPE cartridges of HLB and MCX (Waters, Milford, MA, USA) and OnGuard II A (Thermo Scientific, Sunnyvale, CA, USA) were applied to extract the amino acids in this research. The experimental procedure was based on the operation manual and methods reported in literature [20,21]. For HLB, the cartridge was rinsed with 2 mL of methanol (MeOH) followed by 2 mL of Milli-Q water. Then, the sample that was acidified with 0.2% formic acid (FA) was loaded. Next, 2 mL of MeOH containing 10% Milli-Q water and 0.1% FA was used as washing solution. Subsequently, 500 µL of collected solution was dried and re-dissolved in 1 mL of 30 mM MSA for further analysis. For MCX, the cartridge was rinsed with 2 mL of MeOH followed by 2 mL of 1% FA. The next steps (loading, washing and re-dissolving) were the same as for the HLB cartridge. For OnGuard II A, the cartridge was conditioned with 10 mL of 30 mM MSA, followed by 10 mL of Milli-Q water. Loading 5 mL of sample solution, the first 3 mL of effluent were discarded, and the next 2 mL were collected for analysis. Although some cleanup was realized (see Figure 2), there was still interference, especially with the determination of sodium. Since there are over twenty amino acids in the PN solution, it is hard to totally remove the amino acids from the sample and so attempts using SPE were found to be unsatisfying.

Another approach is to heat the PN solution at a high temperature to decompose the amino acids, while metal ions such as sodium, potassium, magnesium and calcium remain in the residue. According to the European Pharmacopoeia (Ph. Eur.), methods for total ash and sulfated ash are able to remove amino acids from samples [22]. Therefore, the applicability of both methods as such was examined. After calcination, the residues were dissolved in water and injected in the IC system. Both methods provided an efficient cleanup as no further interference with the peaks of interest in the chromatogram was noticed. However, the recovery (Table 1) for Ca^2+^ and Mg^2+^ was below 10% for both procedures. Changing from water to 30 mM MSA as pickup solvent yielded recovery values for Ca^2+^ and Mg^2+^ above 90% (Table 1) with a slightly better performance for the sulfated ash method, while the recovery values for Na^+^ and K^+^ were more than 10% too high. As the H_2_SO_4_ used in this procedure could be a source of Na^+^ and K^+^, concentrated HNO_3_ and MSA, as well as 1 M MSA were also considered as calcination media. The results are presented in Table 1. Finally, 1 M MSA was withheld as the calcination medium with 30 mM MSA as the pickup solution.

**Figure 3:**
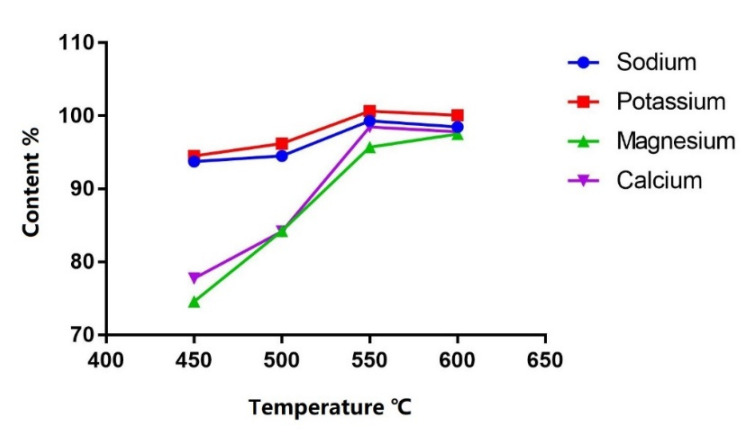
Content (%) of each cation in PN solution after calcination at different temperatures.

Four heating temperatures (450 °C, 500 °C, 550 °C and 600 °C) were compared. Heating at 550 °C for 1 h was the optimal condition considering calcination efficiency as the content tended to be stable from 550 °C and higher (Figure 3).

Concerning the containers, four different types of crucibles were compared, including porcelain, quartz, polytetrafluoroethylene (PTFE) and aluminum oxide (Al-24). For this purpose, Milli-Q water instead of sample solution was used, followed by the addition of 1 M MSA and the calcination procedure described in Section 3.2.2. Porcelain crucibles released considerably more Na^+^, K^+^ and Ca^2+^ compared to quartz crucibles (Table 2). Next, PTFE and Al-24 crucibles were considered. PTFE crucibles were ion free, but the maximum temperature was limited to 270 °C, which is not efficient for calcination. Al-24 crucibles were not useful for liquids because of their porosity. Therefore, quartz crucibles were preferred for calcination since they released only tiny amounts (less than 0.1% of the content in the sample) and showed a sufficiently high temperature tolerance. The final sample pretreatment method for cation analysis has been described in Section 3.2.2.

Next, the influence of glass and plastic vials was examined when they were filled with Milli-Q water or 30 mM MSA. The results indicated that glassware could introduce sodium contamination (Table 3). Therefore, plastic recipients were applied during the whole process of cation analysis.

### 2.2. Method Development and Optimization for Anion Analysis

#### 2.2.1. Anion Analysis Method Optimization

Anions were determined using an IonPac AS19 column. The method for column testing from the manufacturer was applied to set the starting conditions. Therefore, the column temperature was kept at 30 °C and the mobile phase was 20 mM NaOH with a flow rate of 1 mL/min [23]. No contamination was encountered for the determination of chloride. Increasing the temperature to 35 °C resulted in a faster analysis time and also a more stable baseline. As a consequence, we decided to adjust the temperature of the column and detector cell (which are both in the oven compartment) to 35 °C.

#### 2.2.2. Sample Pretreatment for Anion Analysis

As there is no interference with the chloride analysis, a simple dilution step with Milli-Q water was executed before sample injection.

### 2.3. Comparing the Suppressed and Non-Suppressed System

The suppressor is a special device for IC, which is installed after the separation column and is intended to eliminate the highly conductive background and, therefore, enhances the sensitivity of the measured analytes. A system without a suppressor is simpler and cheaper. Even losing some sensitivity, it is still a good choice for ion analysis. The values for the limit of detection (LOD) and limit of quantification (LOQ) of the different analytes are illustrated in Table 4. Finally, the suppressed conductivity method was adopted for determination of inorganic ions in PN since a more stable baseline facilitates integration of the peaks.

### 2.4. Method Validation

#### 2.4.1. Selectivity

No interfering peaks were observed in the blank chromatogram and all peaks were baseline separated in the chromatograms of standard and test sample.

#### 2.4.2. Sensitivity

The measurement of the sensitivity was based on the LOD and LOQ evaluated by the signal-to-noise ratio. The LOD and LOQ values for Na^+^, K^+^, Mg^2+^, Ca^2+^ and Cl^−^ were shown in Table 4.

#### 2.4.3. Linearity

The relationship between peak area (*y*) and concentration of analyte (*x*) was evaluated for Na^+^, K^+^, Mg^2+^, Ca^2+^ and Cl^−^. For all of them, it was found to be linear with determination coefficients above 0.999 (Table 5). The 95% confidence interval of the intercepts included zero for all these regression equations. The residual plots were randomly distributed around the zero axis which indicates that the data fit the linear model well.

#### 2.4.4. Precision

Precision was evaluated for repeatability (3 concentration levels) and inter-day precision as RSD (%). The results are shown in Table 6. The RSD (%) values were not higher than 1.0% for repeatability (*n* = 6) and inter-day precision (*n* = 12), which indicates a good precision for the method.

#### 2.4.5. Recovery

To determine the recovery, Na^+^, K^+^, Mg^2+^, Ca^2+^ and Cl^−^ were added at three different concentration levels (80, 100 and 120 percent of the test sample concentration). The recoveries for these analytes ranged from 99.1% to 101.1% (Table 7), which demonstrates that the method is reliable.

#### 2.4.6. Robustness

**Figure 4:**
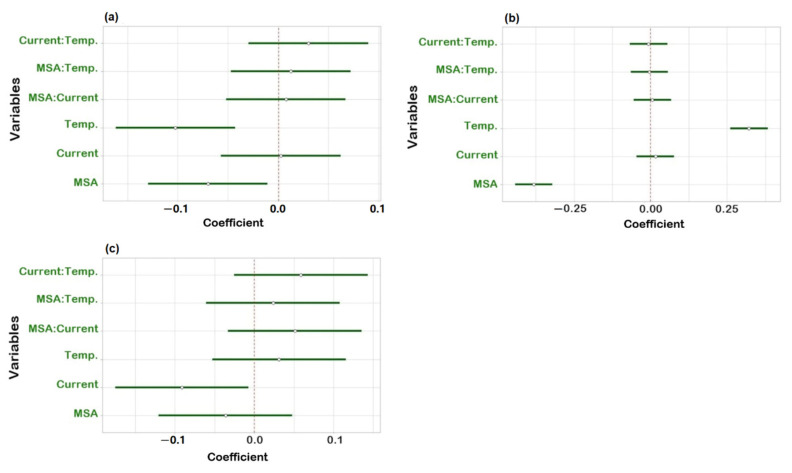
Regression coefficient plots obtained from the robustness study for (**a**) resolution between Na^+^ and K^+^; (**b**) resolution between K^+^ and Mg^2+^; (**c**) resolution between Mg^2+^ and Ca^2+^.

**Figure 5:**
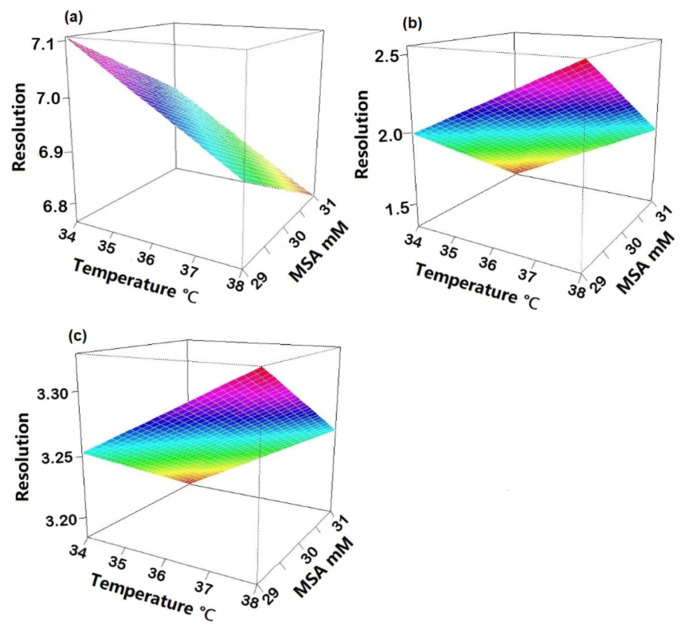
Response surface plots depicting the influence of MSA concentration and temperature on (**a**) resolution between Na^+^ and K^+^; (**b**) resolution between K^+^ and Mg^2+^; (**c**) resolution between Mg^2+^ and Ca^2+^.

To check robustness, an experimental design using R software (version 4.1.3) and R-Studio (Boston, MA, USA) was applied. Three factors were investigated: suppressor current, concentration of MSA in the mobile phase and column temperature. Upon obtaining the experimental results, statistical analysis of the data was carried out to determine the coefficient plots and the response surface plots. No influence was observed on the peak area within the investigated range of the individual variables as all intervals included zero (Figure S1). The three regression coefficient plots shown in Figure 4 illustrate the effect of the individual variables and their interactions on the resolution between Na^+^ and K^+^ (Rs _Na-K_), K^+^ and Mg^2+^ (Rs _K-Mg_) and Mg^2+^ and Ca^2+^ (Rs _Mg-Ca_). In Figure 4a, the concentration of MSA and temperature have a significant negative effect on Rs _Na-K_, meaning that Rs _Na-K_ will decrease when increasing one of those two factors and vice versa. It is shown in Figure 4b that temperature has a positive effect and the concentration of MSA shows a negative effect on Rs _K-Mg_. It can be derived from Figure 4c that the suppressor current has a significant negative effect on Rs _Mg-Ca_. Concerning interactions between two variables, no significant effect was observed as all intervals included zero. Moreover, from the response surface plots (Figure 5), it can be observed that the resolution between sodium and potassium was at least 6.5, the resolution between potassium and magnesium was always above 1.3 and the resolution between magnesium and calcium was over 3.0, under the examined experimental conditions. This means that the developed method is independent of small variations and can be considered robust.

### 2.5. PN Solution Analysis

**Figure 6:**
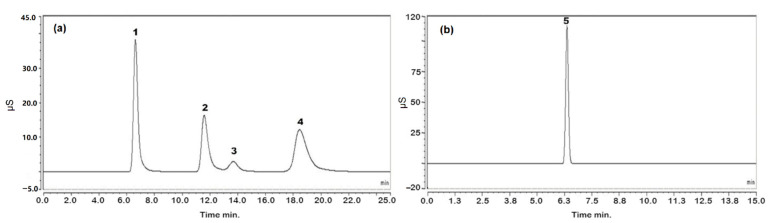
Chromatograms obtained using the optimized conditions (see Section 3.3) for analysis of PN solution: (**a**) chromatogram of cations; (**b**) chromatogram of anions. Peak 1: sodium, peak 2: potassium, peak 3: magnesium, peak 4: calcium and peak 5: chloride.

The contents of the analytes were determined in two different batches of PN solutions. The results are shown in Table 8. The chromatograms obtained following the analysis of PN solution 1 are shown in Figure 6.

## 3. Materials and Methods

### 3.1. Reagents and Materials

Standards are prepared from sodium chloride (99.87%, Merck KGaA, Darmstadt, Germany), potassium chloride (99.5+%, Chem-lab, Zedelgem, Belgium), magnesium chloride (98+%, Sigma-Aldrich, St. Louis, MO, USA) and calcium chloride (97+%, Sigma-Aldrich). MSA (>99.0%, Sigma-Aldrich) and sodium hydroxide (50% *w*/*w* aqueous solution, Acros Organics, Geel, Belgium) were purchased for mobile phase preparation. Water was purified by a Milli-Q water purification system from Millipore (Bedford, MA, USA). The oven (MR 170) was purchased from Heraeus (Hanau, Germany).

Glassware should be avoided for the preparation and storage of solutions and replaced by synthetic material.

### 3.2. Preparation of Standards and Samples

#### 3.2.1. Preparation of Standards

MSA was diluted to 1 M and stored in a plastic bottle at 4 °C as a stock solution. The mobile phase for cation determination was prepared by diluting 1 M MSA to 30 mM MSA in Milli-Q water. Sodium hydroxide (NaOH) was diluted to 20 mM as the mobile phase for anion determination.

Standard stock solutions of the analytes were prepared by dissolving NaCl, KCl, MgCl_2_ and CaCl_2_ in Milli-Q water to obtain concentrations of 22.05 mM for Na^+^, 14.05 mM for K^+^, 1.75 mM for Mg^2+^, 10.50 mM for Ca^2+^ and 4.57 mM for Cl^−^ and stored in plastic bottles at 4 °C for no more than one week. Stock solutions were diluted to the standard solutions ranging from 50 to 150% of the test solution for the linearity tests. Stock solutions of Na^+^, K^+^, Mg^2+^ and Ca^2+^ were mixed and diluted 10 times as 100% standard solution for cation determination. For anion determination, the Cl^−^ stock solution was diluted 2 times as 100% solution.

#### 3.2.2. Preparation and Quantification of Inorganic Ions in PN Solution

The PN solutions were prepared in the hospital pharmacy of UZ Leuven (Leuven, Belgium). The composition of all components is shown in Table 9. A calcination method was developed as a sample pretreatment method for cation analysis: 1.0 mL of PN solution was pipetted in a quartz crucible, then 1 mL of 1 M MSA was added to provide an acidic environment to facilitate calcination. After digesting at 100 °C for 1 h, the residue was heated with a Bunsen burner until white fumes were no longer evolved. Next, the crucible was heated at 550 °C for 1 h and cooled in a desiccator. The residue was dissolved in 20 mL of 30 mM MSA and filtered through a 0.20 µm filter as a test solution for injection. To determine Cl^−^ in the PN solution, the latter was diluted 20 times with Milli-Q water and filtered for injection.

The concentrations of the four cations and the anion in the test solutions were: 50.72, 54.94, 4.20, 41.3 and 81.12 mg/L for Na^+^, K^+^, Mg^2+^, Ca^2+^ and Cl^−^, respectively. All solutions were prepared in duplicate, and each was injected in triplicate.

### 3.3. Instrumentation and Chromatography Conditions

Analyses were conducted using an IC system (ICS-3000, Dionex, Sunnyvale, CA, USA), equipped with a DP analytical pump, an AS50 auto-sampler, a conductivity detector (CD), a Dionex CSRS 300 suppressor for cation determination and a Dionex ADRS 600 suppressor for anion determination. Chromeleon 6.8 was used for data collection and system control.

An IonPac CS16 analytical column (3 mm × 250 mm) from Thermo Scientific (Sunnyvale, CA, USA) was used for cation separation. The eluent was 30 mM MSA at a flow rate of 0.36 mL/min and the column temperature was 36 °C. An IonPac AS19 analytical column (4 mm × 250 mm) from Thermo Scientific was used for anion separation. The eluent was 20 mM NaOH with a flow rate of 1.0 mL/min and the column temperature was 35 °C.

### 3.4. Validation Test

The method was validated for selectivity, sensitivity, linearity, precision, recovery and robustness according to the ICH guidelines [24].

#### 3.4.1. Selectivity

Selectivity was examined by analyzing the standard solution and blank solvent (Milli-Q water) to ensure the separation of the different ions and possible interference from the blank solution consisting of Milli-Q water.

#### 3.4.2. Sensitivity

In order to check the sensitivity of the method under the working conditions used, the LOD and LOQ were determined at a signal-to-noise ratio of 3 and 10, respectively.

#### 3.4.3. Linearity

Calibration curves of different ions were prepared in five concentrations of standard solution ranging from 50 to 150% of the test concentration: 25.36 to 76.07 mg/L for sodium, 27.47 to 82.40 mg/L for potassium, 2.10 to 6.29 mg/L for magnesium, 20.65 to 61.95 mg/L for calcium and 40.56 to 121.68 mg/L for chloride.

#### 3.4.4. Precision

Precision of the IC methods was evaluated by the repeatability and intermediate (inter-day) precision. Repeatability was determined by 6 replicates at 3 levels (80%, 100% and 120% of the test concentrations of the ions) on day 1, while the inter-day precision was evaluated by injecting the 100% solution in triplicate on days 2 and 3.

#### 3.4.5. Recovery

The PN solution is a drug product of which the contents of the ingredients are known. The ingredients were separately available. The recovery of the ions was determined by adding three different concentration levels (80%, 100% and 120% of test concentration) of standard solution to a mixture of components in the PN solution which were not subject of the analysis. The sample pretreatment method was applied for the cations. The recovery was calculated using the following equation:

(1) $$\mathrm{Recovery}\;\left(\%\right)=\frac{\mathrm{experimentally}\;\mathrm{calculated}\;\mathrm{concentration}}{\mathrm{theoretically}\;\mathrm{added}\;\mathrm{concentration}}\times 100$$

#### 3.4.6. Robustness

A robustness test was performed applying an experimental design to ensure the reliability of the analytical method by slightly varying the chromatographic factors within a certain range. In this study, a two-level full factorial design was applied. Three chromatographic parameters (suppressor current, concentration of MSA in the mobile phase and column temperature) were investigated at two levels (−1 and +1) around their central level (0) (Table 10).

According to the experimental design, 2*^k^* experiments were performed in random order, where *k* is the number of factors. Another three experiments with the variables at the central level were carried out at the beginning, middle and end of the series. In total, 11 experiments were carried out in this test. The mathematical relationship between the experimental variables (*x_i_*, *x_j_*, …) and response (*y*) can be obtained from the following equation:

*y* = *b_0_* + *b_i_x_i_* + *b_j_x_j_* + *b_ij_x_i_ x_j_* + ∙∙∙ + *ε* (2)

where *b* are the regression coefficients and *ε* is the experimental error. *b_0_* stands for the intercept, *b_i_* and *b_j_* describe the quantitative effect of the respective variables *x_i_* and *x_j_*, and *b_ij_* represents the interaction effect between both variables. As responses, the resolutions Rs _Na-K_, Rs _K-Mg_ and Rs _Mg-Ca_ as well as the peak areas of Na^+^, K^+^, Mg^2+^, Ca^2+^ and Cl^−^ were selected.

## 4. Conclusions

This is the first report to describe a method for determination of inorganic ions in a PN solution by IC. An accurate, precise and sensitive analytical method was developed and validated for the individual cation and anion determination. Sample pretreatment was found to be necessary to avoid interference from matrix components. An internal standard was not required and would only complicate the procedure.

## Figures and Tables

**Table 1:** Influence of different calcination conditions and pickup solvents on the determination of sodium, potassium, magnesium and calcium.

Calcination	Pickup Solvent	Content %			
		Na^+^	K^+^	Mg^2+^	Ca^2+^
Total ash	water	98	104	6	6
Sulfated ash	water	116	115	9	4
Total ash	30 mM MSA	103	82	95	93
Sulfated ash	30 mM MSA	115	113	97	100
HNO_3_	30 mM MSA	114	115	97	97
MSA	30 mM MSA	107	98	102	103
MSA 1 M	30 mM MSA	104	97	103	100

**Table 2:** Blanks in different crucibles.

	Content mg/L			
	Na^+^	K^+^	Mg^2+^	Ca^2+^
Porcelain crucibles	0.050	<LOQ	<LOQ	0.413
Quartz crucibles	0.005	<LOQ	<LOQ	<LOQ

<LOQ means the content was lower than the LOQ.

**Table 3:** Blanks in different materials.

	Content mg/L			
	Na^+^	K^+^	Mg^2+^	Ca^2+^
Glass vial + water	0.918	0.052	<LOQ	<LOQ
Plastic vial + water	<LOQ	<LOQ	<LOQ	<LOQ
Plastic vial + 30 mM MSA	<LOQ	<LOQ	<LOQ	<LOQ

<LOQ means the content was lower than the LOQ.

**Table 4:** LOD and LOQ of Na^+^, K^+^, Mg^2+^, Ca^2+^ and Cl^−^ obtained with the suppressed and non-suppressed system.

Analyte	Suppressed System		Non-Suppressed System	
	LOD mg/L	LOQ mg/L	LOD mg/L	LOQ mg/L
Na^+^	0.001	0.003	0.1	0.2
K^+^	0.01	0.03	0.3	1.0
Mg^2+^	0.02	0.06	0.2	0.5
Ca^2+^	0.02	0.06	0.3	1.0
Cl^−^	0.0001	0.0002	0.005	0.01

**Table 5:** Regression data for Na^+^, K^+^, Mg^2+^, Ca^2+^ and Cl^−^.

Analyte	Range (mg/L)	Regression Equation	Determination Coefficient
Na^+^	25.36–76.07	*y* = 0.2863 *x* − 0.0726	0.9999
K^+^	27.47–82.40	*y* = 0.1804 *x* − 0.0484	0.9997
Mg^2+^	2.10–6.29	*y* = 0.4374 *x* − 0.0194	0.9997
Ca^2+^	20.65–61.95	*y* = 0.3249 *x* − 0.2192	0.9999
Cl^−^	40.56–121.68	*y* = 0.1077 *x* − 0.1660	0.9992

**Table 6:** Precision of the IC method.

Analyte	Repeatability RSD% (*n* = 6)			Inter-Day Precision RSD% (*n* = 12)
	80%	100%	120%	100%
Na^+^	0.4	0.6	0.5	0.4
K^+^	0.9	0.2	0.5	0.9
Mg^2+^	0.9	0.6	0.3	0.8
Ca^2+^	0.3	0.4	0.7	0.3
Cl^−^	0.8	0.8	0.9	1.0

**Table 7:** Recovery of the IC method.

Analyte	Recovery (%)		
	80%	100%	120%
Na^+^	100.5	100.1	101.0
K^+^	100.3	99.1	100.9
Mg^2+^	99.4	101.1	99.5
Ca^2+^	100.4	100.1	100.2
Cl^−^	100.5	99.7	99.9

**Table 8:** Contents (RSD%, *n* = 6) of different ions following analysis of PN solutions.

PN Solution	Na^+^ (%)	K^+^ (%)	Mg^2+^ (%)	Ca^2+^ (%)	Cl^−^ (%)
1	102.4 (0.7)	99.4 (0.7)	101.2 (0.5)	101.3 (0.8)	99.1 (0.8)
2	100.4 (0.6)	98.9 (0.7)	100.8 (0.9)	100.1 (0.5)	100.5 (0.9)

**Table 9:** Composition of the PN solution.

Ingredients	Contents
Sodium glycerophosphate	Sodium 23.8 mM
K_2_HPO_4_ anhydrous	Potassium 8.6 mM
KH_2_PO_4_ anhydrous	Potassium 1.0 mM
Ca gluconate 10%	Calcium 20.65 mM
KCl	Potassium 18.5 mM, chloride 18.5 mM
NaCl	Sodium 20.3 mM, chloride 20.3 mM
MgCl_2_ 6 aqua	Magnesium 3.45 mM, chloride 6.9 mM
Vaminolact	Alanine 6.3 g/L, arginine 4.1 g/L, aspartic acid 4.1 g/L, cysteine/cystine 1.0 g/L, glutamic acid 7.1 g/L, glycine 2.1 g/L, histidine 2.1 g/L, isoleucine 3.1 g/L, leucine 7.0 g/L, lysine 5.6 g/L, methionine 1.3 g/L, phenylalanine 2.7 g/L, proline 5.6 g/L, serine 3.8 g/L, taurine 0.3 g/L, threonine 3.6 g/L, tryptophan 1.4 g/L, tyrosine 0.5 g/L, valine 3.6 g/L
Glucose 70% Water for injection	Glucose 70% 240 mL/L

**Table 10:** Chromatographic parameter settings applied in the experimental design of the robustness study.

Parameter	Low Value (−)	Central Value (0)	High Value (+)
Suppressor current (mA)	30	32	34
Concentration of MSA (mM)	29	30	31
Column temperature (°C)	34	36	38

## Data Availability

According to policy of KU Leuven. Authors may be contacted for more details concerning data supporting reported results.

## Supplementary Materials

The following supporting information can be downloaded at: https://www.mdpi.com/article/10.3390/molecules27165266/s1, Figure S1: Regression coefficient plots obtained from the robustness study for (a) peak area of Na^+^; (b) peak area of K^+^; (c) peak area of Mg^2+^; (d) peak area of Ca^2+^; (e) peak area of Cl^−^.
