# Supplementary material for: Determination of Inorganic Ions in Parenteral Nutrition Solutions by Ion Chromatography
Source: Molecules. 2022 Aug 18;27(16):5266. doi: 10.3390/molecules27165266 (PMC9416023; doi:10.3390/molecules27165266)
Supplement: Supplementary file 1 [file molecules-27-05266-s001.zip › molecules-1792750-supplementary.pdf]

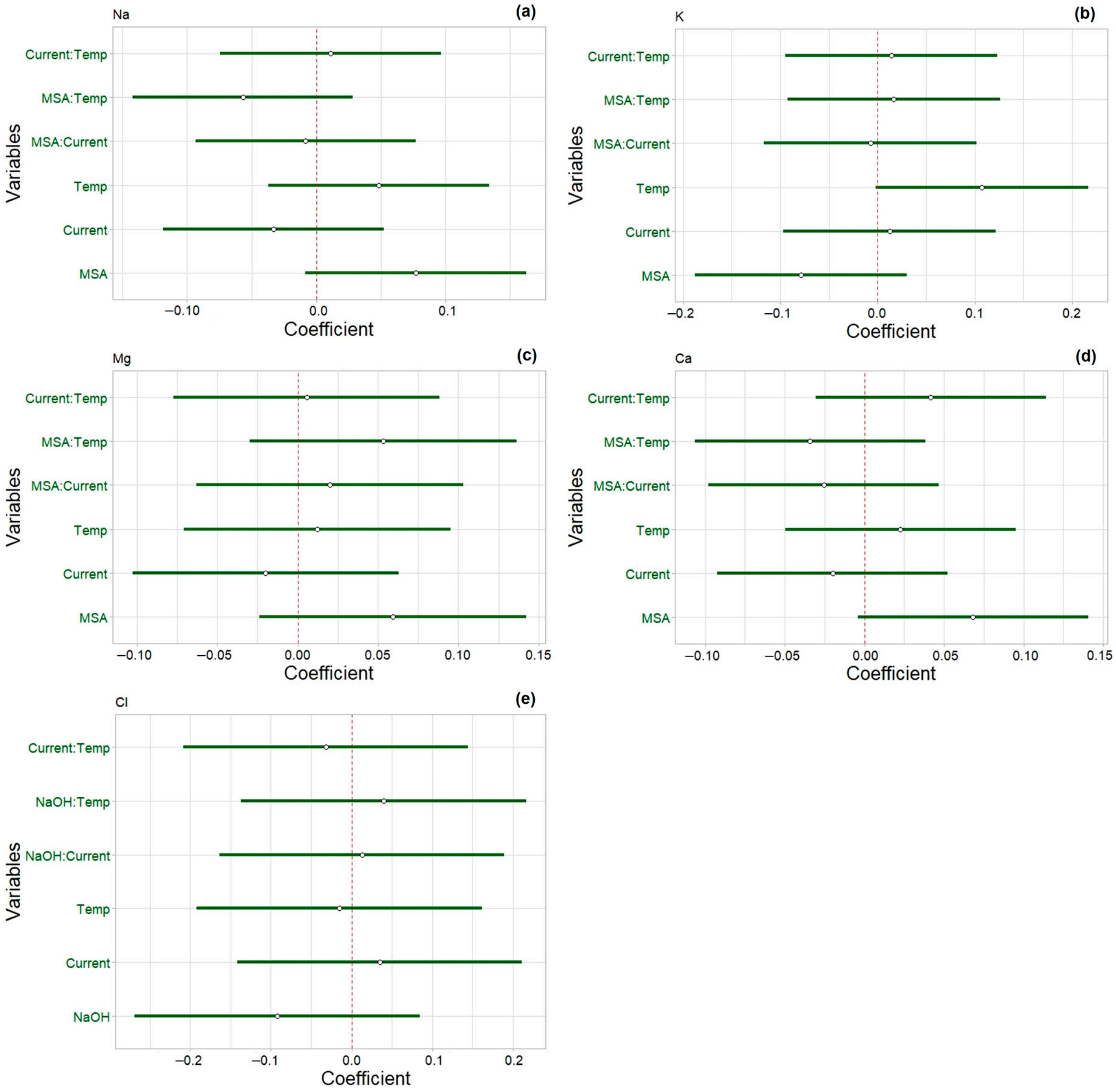

Figure S1: Regression coefficient plots obtained from the robustness study for (a) peak area of Na^{+}; (b) peak area of K^{+}; (c) peak area of Mg^{2+}; (d) peak area of Ca^{2+}; (e) peak area of Cl^{-}.
